# Assessment of Anthelmintic Resistance in Major Gastrointestinal Nematodes in Sheep and Goat Farms in Mymensingh, Bangladesh

**DOI:** 10.1155/japr/5515786

**Published:** 2026-06-22

**Authors:** Md. Rakibul Islam, Md. Mehedi Hasan Mizan, Anisuzzaman, Mst. Sawda Khatun, Faishal Al Ashad, Mohammad Zahangir Alam, Babul Chandra Roy, Anita Rani Dey

**Affiliations:** ^1^ Department of Parasitology, Bangladesh Agricultural University, Mymensingh, Bangladesh, bau.edu.bd

**Keywords:** anthelmintic resistance, EHA, FECRT, goats, sheep

## Abstract

Anthelmintic resistance (AR) is a well concern in livestock production. Hence, the study was designed to assess the status of AR in sheep and goat farms at Mymensingh sadar using fecal egg count reduction test (FECRT) and egg hatch assay (EHA). To do this, 10 farms were selected. For FECRT, 40 animals of each farm having more than 200 EPG of feces were selected randomly by employing the McMaster technique and treated with albendazole (ABZ), levamisole (LEV), and ivermectin (IVM). Fecal samples from animals of each treated and control group were collected directly from the rectum on Day 0 (pretreatment) and Day 14^th^ posttreatment (p.t.), pooled, and considered for coproculture to detect resistant parasites. For EHA, pooled fecal samples were collected from each farm and isolated eggs by combining flotation and sedimentation techniques. The isolated eggs were treated with different concentrations of ABZ solution, such as 0.05, 0.1, 0.2, 0.3, and 0.5 *μ*g/mL, by maintaining a control group with no ABZ solution. The result revealed that in FECRT, ABZ resistance against gastrointestinal (GI) nematodes was detected in nine farms and suspected resistance in one farm. LEV resistance was detected in six farms and suspected resistance in four farms. Interestingly, IVM resistance was developed in one farm, suspected resistance in five farms, and susceptible against GI nematodes in four farms. Also, *Haemonchus* and *Oesophagostomum* were recognized as resistant parasites using coproculture. In EHA, ABZ resistance was also detected in all farms. The EC_50_ value of ABZ ranged from 0.1290 to 0.2393 *μ*g ABZ/mL (> 0.1 *μ*g/mL) with the coefficient of correlation (*R* ^2^) > 0.98. The present study suggests that AR is increasing and creates an upsetting situation in controlling GI nematodes in Bangladesh. Therefore, the use of alternative control methods such as ethnomedicine, bioactive forages, and selective treatment procedure should be practiced.

## 1. Introduction

Gastrointestinal (GI) nematode infections are the most prevalent and pivotal problem affecting small and large ruminants around the world [1]. The control and treatment of parasitic infections have mainly been based on anthelmintics over the last four decades, and benzimidazoles (BZs), macrocyclic lactones (MLs), tetrahydropyrimidines‐imidazothiazoles, aminoacetonitrile derivatives (TAADs), and spiroindoles are commonly used anthelmintics against a wide range of nematode parasites [2]. Dose, treatment frequency, mode of administration, and prolonged use of the same anthelmintics are the key factors governing the development of anthelmintic resistance (AR) that affects the control of parasitic infections [3]. AR was reported first in *Haemonchus contortus* against BZ in 1964 in sheep and rapidly developed in other nematodes affecting cattle and horses [4] in different parts of the world [5–7]. The highest reports on AR have been recorded for ivermectin (IVM) and albendazole (ABZ) or fenbendazole (FBZ), followed by levamisole (LEV) and to some extent moxidectin against nematodes. Among nematodes, resistance against *H. contortus* was reported for all classes of anthelmintics [8]. In addition to *H. contortus*, resistance has developed in *Teladorsagia circumcincta*, *Trichostrongylus colubriformis*, *Oesophagostomum*, *Ostertagia*, and *Cooperia*, in Australia, New Zealand, South Africa, many European countries, several Asian countries, and both American continents [9]. Recently, multispecies and multidrug anthelmintic resistance (mAR) in many countries is an emerging problem and spreading rapidly [10–12]; thus, threatening the sustainability of farm animal production, especially small ruminant farming [13, 14]. The profitability and sustainability of livestock farming is greatly hampered or has failed to achieve its optimum level due to AR or mAR. AR signifies major economic losses in livestock by reducing the effectiveness of treatment, decreasing animal productivity, that is, milk and meat, increasing treatment costs (using more drugs), and higher mortality. The global feature shows about €1.8 billion and $436 million annual loss from parasitic infections in livestock, respectively, in Europe and Australia. Resistance also decreases carcass weight and quality and finally the sale value of animals [15].

Early detection of AR helps to mitigate the spreading of resistant parasite. Various methods have been adopted to assess the level of AR of strongylid nematodes in small ruminants. The in vivo technique such as fecal egg count reduction test (FECRT) and in vitro technique such as egg hatch assay (EHA) are the extensively used techniques for the detection of AR [16, 17]. FECRT is a gold standard test recommended by the World Association for the Advancement of Veterinary Parasitology (WAAVP), and EHA is a commonly used technique for the detection of BZ resistance [9].

A wide range of parasites are prevalent in Bangladesh because of suitable environmental condition for free living stage of parasites [18]. The government of Bangladesh allocates a major portion of drugs (around 37% anthelmintics) for the treatment purpose of affected animals. However, the burden of helminth infection is unchanged or even increased in some cases, indicating development of AR or mAR [16]. First report on AR against GI nematodes with ABZ has been documented in three dairy farms in different areas of Mymensingh, Bangladesh in 2003 [19]. After a long duration in 2020, AR was reported only in one goat farm from Mymensingh using FECRT and EHA [16]. Mymensingh, a big area covering 4363 km^2^ and 40 lakh livestock population remained almost unevaluated. In our previous study, only few (six) government sheep or goat farms established in six different districts were evaluated. Our findings on AR encouraged us to conduct studies in the major livestock population reared in nongovernment farms as well. Therefore, the present study was aimed at evaluating the present status of AR covering eight goat and two sheep farms containing a large number of sheep and goats at Mymensingh sadar using FECRT and EHA.

## 2. Materials and Methods

### 2.1. Study Area

**Figure 1:**
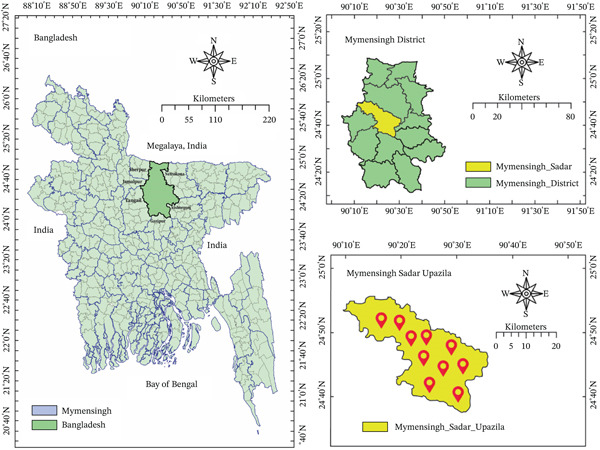
Eight goats and two sheep farms at Mymensingh Sadar Upazila, Mymensingh, Bangladesh where the experiments were conducted.

The study was conducted at eight goats and two sheep farms at Mymensingh Sadar Upazila, Mymensingh, Bangladesh (Figure 1). The number of animals in each farm varied from 70 to 100. The farms were managed in a semi‐intensive system, involving grazing on roadside grasses and tree leaves, and supplemented by homemade concentrates like rice polish and oil cakes. Elevated slatted‐floor houses made of wood/bamboo with tin sheds were practiced in the studied farms. Cleaning and drinking water were supplied from tube wells and ponds. Usually, natural breeding was practiced with local bucks, and only PPR vaccine was used in these farms. According to the record book, parasitic infections were prevalent in these farms and for deworming ABZ, LEV, and IVM were used. Interestingly, farmers used anthelmintics for their animals without maintaining proper dose rates, and they do not have any fixed deworming schedule.

### 2.2. Selection of Farms and Animals, and Examination of Samples

The farms were selected based on the ease of obtaining permission to collect sample from the respective farms. These farms are away from highway and protected from street dog, cat, and other wild animals. At least 60 animals of both sexes and comparable age (6 months–2 years and 6 months) from each farm were randomly selected to collect fecal samples. Fecal samples were examined using McMaster technique as described previously [18]. Briefly, 2 g of fecal materials was mixed with 28‐mL flotation solution (saturated salt solution, specific gravity: 1.200). The suspension was stirred gently and sieved to remove coarse particles. The chambers of McMaster slide were filled with the suspension and allowed to stand for 5 min to float parasitic eggs. Then, the slide was examined under microscope (LABOMED, Labo America, Inc., United States) using low power objective (10×) and counted all eggs within the etched grid lines of the chambers. The total numbers of eggs were multiplied by 50; thus, the calculation gave the eggs per gram of feces (EPG). After calculating EPG, animals with at least 200 EGP and treated with anthelmintics at least 3 months before were included; otherwise, they were excluded from this study. Finally, 40 animals (sheep or goats) from each farm and a total of 400 animals (320 goats and 80 sheep) were selected from eight goat and two sheep farms.

### 2.3. Fecal Egg Count Reduction Test (FECRT)

The selected 40 animals from each farm were weighed using a digital weighing machine and divided into four groups (three treatment groups and one control group). During grouping, animals were randomized and each group consisted of 10 animals. The in vivo experiments were conducted strictly following the ARRIVE guidelines (https://arriveguidelines.org/resources/author-checklists). Group 1 was treated with ABZ (at 7.5 mg/kg body weight oral administration), Group 2 with LEV (at 7.5 mg/kg body weight oral administration), Group 3 with IVM (at 0.2 mg/kg body weight subcutaneous injection). Oral treatment was administered using a syringe to ensure dosing accuracy. Animals belonging to Group 4 received no medication and served as control. Fresh fecal samples from each animal were collected at Day 0 (pretreatment) and Day 14 (posttreatment [p.t.]), and EPG was calculated by employing the McMaster technique [16]. The Day‐14 interval was chosen for all groups to ensure consistency and comparability as per the guidelines of WAAVP [20].

For the detection of resistant larvae, fecal samples were collected from controls and treatmentgroups separately at Day 0 (pretreatment) and Day 14 (p.t.) from each farm. A total of 50 g of fecal samples, almost similar amount of samples from each animal in each group were collected, pooled group wise, placed separately in glass petri dishes and covered with a polythene sheet. Then the petri dish was kept at room temperature (22°C–27°C) at 80% humidity for 7 days. During the incubation period, water was sprayed to maintain moisture level [20]. After 7 days, accumulated larvae on the contact surface of the polythene were collected by washing three times with normal saline and centrifuged. Larvae were examined under a microscope by adding a drop of Lugol′s iodine and were identified third stage larvae (L3) according to the morphological features given by Van Wyk et al. [21].

### 2.4. Egg Hatch Assay (EHA)

EHA was performed to detect ABZ resistance according to Dey et al. [16]. In brief, 50 g of pooled fecal samples from each farm were kept in a 100‐mL glass bottle, mixed with water, and the entire environment was made anaerobic by thorough shaking. The combination of flotation and centrifugation techniques was used to isolate eggs from the pooled samples and made a suspension with a desired concentration of egg (100–150 eggs/100 *μ*L).

### 2.5. Isolation of Eggs From Pooled Samples

The collected pooled samples were sieved to remove coarse particles, centrifuged, and gently poured off the supernatant. Then, the sediment was resuspended with saturated salt solution and centrifuged. The supernatant was collected carefully in a conical glass centrifuge tube, filled with distilled water, and centrifuged again. Finally, the supernatant was removed and sediment was examined, and EPG was calculated by McMaster technique. The final concentration of egg was adjusted to 100–150 eggs/100 *μ*L of suspensions.

### 2.6. Preparation of Different Concentration of ABZ

For the preparation of stock solution, 50 mg ABZ was dissolved in 5‐mL dimethyl sulfoxide (DMSO). To perform EHA, 0.05, 0.1, 0.2, 0.3 and 0.5 *μ*g of ABZ per mL was used with the final volume of 2000 *μ*L in a 24‐well culture plate. The same volume of egg suspension and drug suspension was used in each well. In the control well, only 0.5% DMSO was used. Each test was performed in triplicate. The culture plate was incubated at 25°C for 48 h in a CO_2_ incubator (Biobase Bioindustry Co. Ltd., Shandong, China) and then the development of eggs was stopped with Lugol′s iodine. Each well was examined under a microscope (10X magnification) in a blinded manner. During examination, at least 100 eggs and/or larvae were counted from each well [22].

### 2.7. Statistical Analysis

#### 2.7.1. FECRT

The percentage reduction in fecal egg count and 95% confidence interval were calculated using the arithmetic mean of egg counts according to the guidelines of WAAVP. The formula for determining the percentage of reduction was 100(1‐Xt/Xc), where “Xt” is the mean egg count of the treated group, and “Xc” is the mean egg count of the control group. AR was considered if the percentage reduction in egg count was less than 95%, and the lower limit for its 95% confidence interval was equal to or less than 90%; and suspected resistance when only one of these two criteria was met out.

#### 2.7.2. EHA

Using the computer software GraphPad Prism 9.5 for Windows, a four‐parameter logistic equation with a variable slope was selected to fit the achieved dose‐response data by nonlinear regression. After converting the drug concentrations to their logarithm (*X* = logX) and setting the bottom number to 0, the EC_50_ values, 95% confidence intervals, and *R* ^2^ values were computed. The EC_50_ value more than 0.1 *μ*g/mL was considered as ABZ resistance [20]. Graphs were also prepared in GraphPad Prism.

## 3. Results

### 3.1. Development of mAR in Sheep and Goat Farms Assessed by FECRT

In this study, the status of AR in eight goat and two sheep farms was assessed from the results of percentage of fecal egg reduction and 95% confidence intervals based on the principles suggested by WAAVP. The result revealed that AR had developed to one or more classes of anthelmintics in almost all the farms. AR was estimated against GI nematodes for ABZ, LEV and IVM in all farms. ABZ resistance against GI nematodes was detected in nine farms and suspected resistance in one farm. LEV resistance was detected in six farms and suspected resistance in four farms. Interestingly, IVM resistance had developed in one farm, suspected resistance in five farms, and four farms were susceptible against GI nematodes. Among the eight goat farms, ABZ and LEV resistance or suspected resistance against GI nematodes had developed in all eight farms, whereas IVM resistance or suspected resistance against GI nematodes was found in five goat farms. In the case of the two sheep farms, ABZ and LEV resistance or suspected resistance was detected in both sheep farms, but IVM suspected resistance against GI nematodes was found in one sheep farm (Table 1).

**Table 1:** FECRT results measuring the level of anthelmintic resistance in eight goats and two sheep farms in Mymensingh Sadar Upazila.

Anthelmintic used	Goat farms				Sheep farms			
	Total no. of farms	No. with resistant (%) with FECR	No. with suspected resistant (%) with FECR	No. with susceptible (%) with FECR	Total no. of farms	No. with resistant (%) with FECR	No. with suspected resistant (%) with FECR	No. with susceptible (%) with FECR
**ABZ**	8	7 (88),	1 (12),	0 (0)	2	2 (100),	0 (0)	0 (0)
		86.84–94.44	96.44			93.75–94.14		
**LEV**	8	5 (63),	3 (37),	0 (0)	2	1 (50),	1 (50),	0 (0)
		83.55–93.98	94.91–95.37			90.62	96.07	
**IVM**	8	1 (12),	4 (50),	3 (38),	2	0 (0)	1 (50),	1 (50),
		93.42	95.74–97.68	97.58–98.82			96.87	98.04

Abbreviations: ABZ, albendazole; IVM, ivermectin; LEV, levamisole; R, resistance; S, susceptible; SR, suspected resistance.

### 3.2. *Haemonchus* and *Oesophagostomum* Were Resistant Larvae in Studied Farms

**Figure 2:**
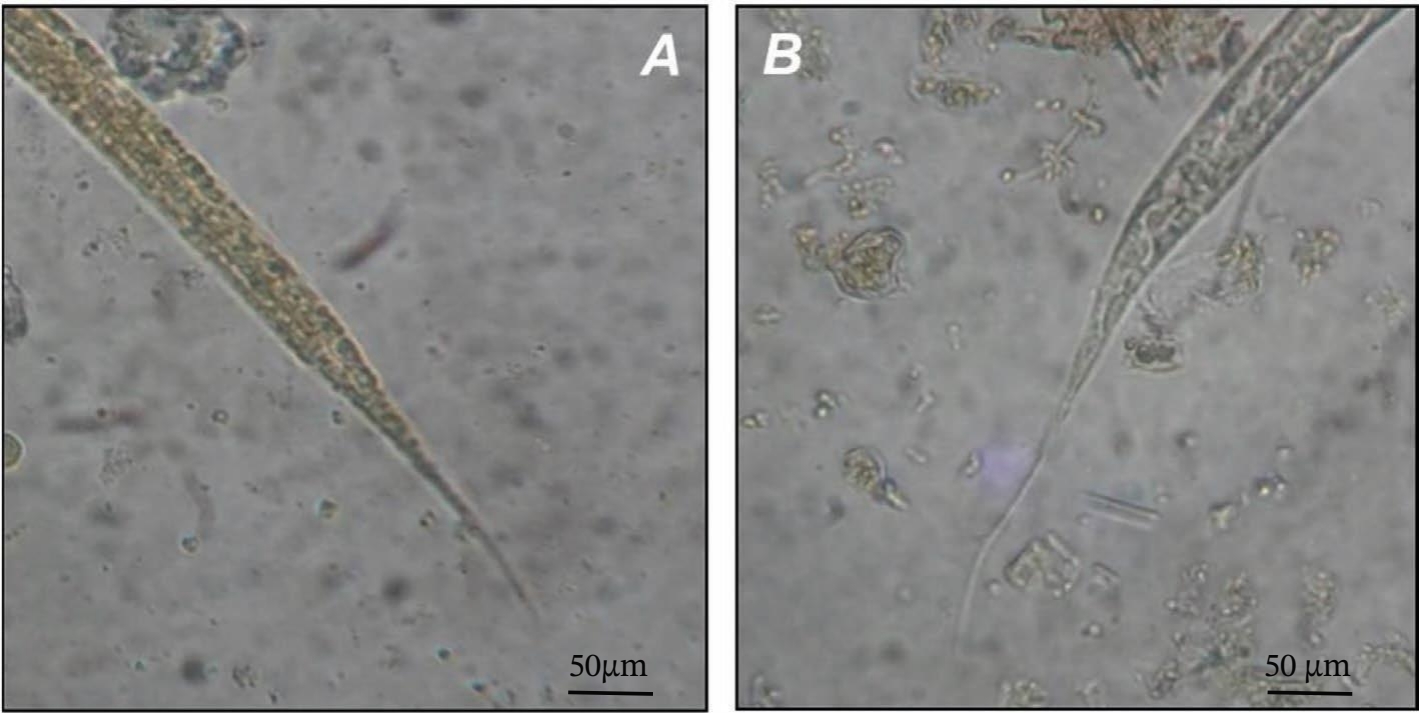
Detected resistant larvae isolated from fecal culture at Day 14 of post treatment. Larvae of (A) *Haemonchus* and (B) *Oesophagostomum* were identified by their characteristic extended tail sheath.

To detect specific resistant nematodes, pooled fecal samples from each group were evaluated. Before treatment, we detected *Haemonchus, Oesophagostomum, Trichostrongylus* and *Strongyloides*. However, at Day 14 of p.t., L_3_ of *Haemonchus* and *Oesophagostomum* were recovered from fecal culture of *Haemonchus* and *Oesophagostomum* isolated from culture were identified by their key morphological features. The L_3_ of *Haemonchus* and *Oesophagostomum* were slender and almost similar in size; however, extension of tail sheath beyond the tail of larvae varied and it was more than double in *Oesophagostomum* (Figure 2). *Haemonchus* and *Oesophagostomum* were recovered from seven farms and in three farms only *Haemonchus* was detected (Table 2).

**Table 2:** The recovered larvae from coproculture in 10 sheep and goat farms at Day 0 (pretreatment) and Day 14 (posttreatment).

No. of farms	Before treatment in culture (Day 0)	After treatment in culture (Day 14)
Goat Farm 1	*Haemonchus* spp.	*Haemonchus* spp.
	*Oesophagostomum* spp.	*Oesophagostomum* spp.
	*Trichostrongylus* spp.	
	*Strongyloides* spp.	

Goat Farm 2	*Haemonchus* spp.	*Haemonchus* spp.
	*Oesophagostomum* spp.	*Oesophagostomum* spp.
	*Strongyloides* spp.	
	*Trichostrongylus* spp.	

Goat Farm 3	*Haemonchus* spp.	*Haemonchus* spp.
	*Oesophagostomum* spp.	
	*Trichostrongylus* spp.	

Goat Farm 4	*Haemonchus* spp.	*Haemonchus* spp.
	*Oesophagostomum* spp.	*Oesophagostomum* spp.
	*Strongyloides* spp.	
	*Trichostrongylus* spp.	

Goat Farm 5	*Haemonchus* spp.	*Haemonchus* spp.
	*Oesophagostomum* spp.	*Oesophagostomum* spp.
	*Trichostrongylus* spp.	

Goat Farm 6	*Haemonchus* spp.	*Haemonchus* spp.
	*Oesophagostomum* spp.	*Oesophagostomum* spp.
	*Strongyloides* spp.	
	*Trichostrongylus* spp.	

Goat Farm 7	*Haemonchus* spp.	*Haemonchus* spp.
	*Oesophagostomum* spp.	
	*Trichostrongylus* spp.	

Goat Farm 8	*Haemonchus* spp.	*Haemonchus* spp.
	*Trichostrongylus* spp.	
	*Oesophagostomum* spp.	

Sheep Farm 1	*Oesophagostomum* spp.	*Haemonchus* spp.
	*Strongyloides* spp.	*Oesophagostomum* spp.
	*Trichostrongylus* spp.	
	*Haemonchus* spp.	

Sheep Farm 2	*Haemonchus* spp.	*Haemonchus* spp.
	*Strongyloides* spp.	*Oesophagostomum* spp.
	*Oesophagostomum* sp.	

### 3.3. Confirmation of ABZ Resistance in Sheep and Goat Farms Using EHA

We estimated that the EC_50_ value of ABZ in all studied farms ranged from 0.1290 to 0.2393 *μ*g/ml. According to WAAVP, the EC_50_ value > 0.1 *μ*g ABZ/mL indicates resistance. By the following recommendation, resistance to ABZ against GI nematodes had been detected in all studied farms. The coefficient of correlation (*R* ^2^) was above 0.98 in all cases. Additionally, the coefficient of correlation *R* ^2^ value above 0.98 indicates that there is a strong correlation between the dose‐response relationship of the drug and the observed effects on the parasites (Table 3).

**Table 3:** The EC_50_ value of egg hatch assay in eight goat and two sheep farms at Mymensingh Sadar Upazila.

Name of farms	*E* *C*_50_(*μ*g/mL)	95% CI	*R* ^2^
Goat Farm 1	0.2228	0.1875–0.2875	0.9894
Goat Farm 2	0.2393	0.1958–0.2930	0.9921
Goat Farm 3	0.1456	0.1132–0.2337	0.9994
Goat Farm 4	0.1984	0.1576–0.3088	0.9995
Goat Farm 5	0.1717	0.1295–0.3308	0.9988
Goat Farm 6	0.1290	0.1194–0.1409	0.9999
Goat Farm 7	0.1376	0.0924–0.6091	0.9980
Goat Farm 8	0.1694	0.1261–0.3536	0.9982
Sheep Farm 1	0.1558	0.1355–0.1869	0.9997
Sheep Farm 2	0.1687	0.1331–0.2609	0.9990

Abbreviations: CI, confidence interval; EC_50_, half maximal effective concentration; *R* ^2^, correlation coefficient.

## 4. Discussion

AR in GI nematodes is a serious concern to the health and welfare of livestock. It has been reported at farm level [23] as well as in naturally grazing animals [24, 25] over the world [8], but most frequently detected in small ruminants [26, 27]. Globally, the profitable and sustainable livestock productions are at risk because of increasing the prevalence of AR against the prevalence of helminth populations [28].

AR is a growing problem and prevalent in most of the African countries, particularly in livestock production where resistance to all major classes of anthelmintics (BZs, LEV, and MLs) has been extensively reported [29]. It is also a serious issue in Asia in GI nematode populations, which have been documented in China, India, Pakistan, and Iran [30]. In the United Kingdom, AR has been reported in GI nematodes and also in other groups of parasites [25].

In the current study, FECRT was performed in eight goat and two sheep farms to assess the status of AR and AR had been detected in all farms ranging from single or multiple drug resistance. ABZ and LEV resistance or suspected resistance were detected in 100% of farms, whereas IVM resistance was found in 60% of farms. The variation of resistance status in different farms might be due to variation in management practices, deworming, and quality of drug used [31, 32]. ABZ and LEV resistance against GI nematodes in small ruminants have also been reported in other parts of the world [33–37]. On the other hand, IVM has been shown to be an effective drug throughout the globe [38] including Bangladesh [39, 40], but AR against this drug has also been documented [41].

The status of AR against GI nematodes varied by regions, classes of anthelmintics, and host species. In Norway, ABZ resistance has been detected in 11% of sheep farms, whereas IVM, BZ, and LEV resistance were detected in 23%, 3.7%, and 7.4% of farms, respectively; in Slovakia, BZ and LEV resistance were detected in 83% and 50% of sheep farms, respectively; and in Western France, similar resistance trends were observed [25]. In Malaysia, among 39 sheep and 9 goat farms, AR was diagnosed in 94.9% of sheep and 77.8% of goat farms. Of the available used anthelmintics, the highest level of resistance has been detected in BZ (50%) followed by LEV (23%), IVM (7%) and closantel (7%) in sheep farms. In goat farms, closantel resistance has been detected at the highest level (100%) followed by BZ (75%) and LEV (57%). However, moxidectin is susceptible in both sheep and goat farms [23]. Bioclimatic variables, farm management, application of anthelmintic treatment and the survival of nematodes in refugia might be the important factors for the development of AR.

GI nematodes are the most prevalent helminth in different geographical regions of Bangladesh [18]. In this study, *Haemonchus*, *Oesophagostomum*, *Trichostrongylus* and *Strongyloides* were found available in the studied farms before treatment, and *Haemonchus* and *Oesophagostomum* were detected at 14 days p.t. from fecal culture. *Haemonchus* and *Trichostrongylus* species have been identified as resistant parasites in sheep and goats, primarily in tropical and subtropical areas. From the study of Alaro et al. [42], *Haemonchus*, *Trichostrongylus*, and *Strongyloides* are found as resistant parasites in naturally‐infected goats in Southern Ethiopia. *Haemonchus* is reported as the highly abundant resistant nematode along with *Trichostrongylus*, *Teladorsagia*, *Cooperia*, and *Oesophagostomum* in different geographical areas of the world due to higher ecological and biological adaptability [34, 43–48]. Among different nematodes, *Haemonchus* shows very high reproductive potentials, genetic diversity and gene flow; thus, it helps prevent the anthelmintic effects and disseminate the resistant gene [49].

Estimated EC_50_ value in the present study revealed ABZ resistance in all farms with varying levels of resistance. EHA is suitable and only used in vitro technique for the diagnosis of BZ resistance [22]. EHA can diagnose the status of resistance when >25% of a nematode population contains the resistance allele [50]. Several authors used this test for the diagnosis of BZ resistance at field level in sheep and goats [9, 16, 51–55].

The development of AR in parasite populations can be restricted by administering proper dose and the frequency and timing of anthelmintic treatment. Estimation of proper dose depends on calculation of accurate weight of the animals. However, at field level, the effective dose is determined by visual appraisal of an animal to assess its weight, which is frequently deviated from actual weight, thus affects effective dosing. This under dosing in turn elicits the development of AR [44].

In conclusion, AR was prevalent in all goat and sheep farms against GI parasites where ABZ resistance or suspected resistance was most prevalent (all 10 farms) followed by LEV (all 10 six farms) and least with IVM (six farms). Notably, IVM was susceptible against GI nematodes in four farms. *Haemonchus* and *Oesophagostomum* were identified as the resistant nematodes. The result of EHA also revealed ABZ resistance in all ten farms and the EC_50_ value of ABZ ranged from 0.1290 to 0.2393 *μ*g/mL with the coefficient of correlation (*R* ^2^) > 0.98. Taken together, our study revealed that improvement of farm management is required particularly through pasture management and rotation of grazing, if possible. Also, both strategic deworming and fecal egg count–based deworming program should be introduced at farm level.

## Funding

This study was supported by the University Grants Commission, Bangladesh (2023/13/UGC).

## Ethics Statement

No animals were harmed or unethically injured/killed. The study was approved by the Animal Welfare and Ethical Committee of Bangladesh Agricultural University (06/AWEC/2017).

## Conflicts of Interest

The authors declare no conflicts of interest.

## Data Availability

The data that support the findings of this study are available from the corresponding author upon reasonable request.
